# Animal models of human atherosclerosis: current progress

**DOI:** 10.1590/1414-431X20209557

**Published:** 2020-05-18

**Authors:** A.V. Poznyak, Y.Y. Silaeva, A.N. Orekhov, A.V. Deykin

**Affiliations:** 1 Institute for Atherosclerosis Research, Skolkovo Innovative Center, Moscow, Russia; 2 Core Facility Centre, Institute of Gene Biology, Russian Academy of Sciences, Moscow, Russia; 3 Laboratory of Angiopathology, Institute of General Pathology and Pathophysiology, Moscow, Russia; 4 Institute of Human Morphology, Moscow, Russia; 5 Center for Precision Genome Editing and Genetic Technologies for Biomedicine, Institute of Gene Biology, Russian Academy of Sciences, Moscow, Russia

**Keywords:** Atherosclerosis, Animal models, Genetically engineered model animals, Rodents

## Abstract

Atherosclerosis retains the leading position among the causes of global morbidity and mortality worldwide, especially in the industrialized countries. Despite the continuing efforts to investigate disease pathogenesis and find the potential points of effective therapeutic intervention, our understanding of atherosclerosis mechanisms remains limited. This is partly due to the multifactorial nature of the disease pathogenesis, when several factors so different as altered lipid metabolism, increased oxidative stress, and chronic inflammation act together leading to the formation and progression of atherosclerotic plaques. Adequate animal models are currently indispensable for studying these processes and searching for novel therapies. Animal models based on rodents, such as mice and rats, and rabbits represent important tools for studying atherosclerosis. Currently, genetically modified animals allow for previously unknown possibilities in modelling the disease and its most relevant aspects. In this review, we describe the recent progress made in creating such models and discuss the most important findings obtained with them to date.

## Introduction

Atherosclerosis lies at the basis of severe human diseases that account for a large part of global morbidity and mortality, such as ischemic heart disease, myocardial infarction, and stroke. This disease can affect any artery in the human body, but is especially dangerous in large vital vessels, such as the carotid and coronary arteries. Certain parts of blood vessels that have bends or bifurcations are more susceptible to atherosclerotic plaque formation. Growing atherosclerotic plaque can by itself reduce the vessel volume leading to pathological consequences for the alimented organ or tissue. However, thrombotic events that occur at the surface of so-called unstable plaques are more dangerous. Thrombosis associated with rupture or erosion of atherosclerotic plaques is the cause of many cases of sudden cardiac death (1).

According to current understanding, atherosclerosis is a multifactorial disease that involves altered lipid metabolism, increased oxidative stress, impaired mitochondrial function, and chronic inflammation (2,3). The initial stages of atherosclerotic plaque development take place at the surface of the blood vessel and include local disturbance of endothelial function. Activation of endothelial cells leads to increased permeability of the endothelium for circulating lipids and to the recruitment of patrolling immune cells. Both innate and adaptive immune responses take part in this process (4,5). The developing atherosclerotic lesion is associated with increased entry and subsequent accumulation of atherogenic lipoproteins in the sub-endothelial space of the arterial wall, the intima-media layer (6,7). This process is followed by massive intracellular accumulation of lipids by migrated cells, both recruited immune cells and migrated resident arterial wall cells, such as pericytes and vascular smooth muscular cells (VSMCs) that alter their phenotype to acquire the ability for phagocytosis. The so-called foam cells, with cytoplasm filled with lipid droplets, are common constituents of growing atherosclerotic plaques. The advanced plaques have a prominent lipid core that can contain a necrotic core formed through uncontrolled cell death and deficient clearance. Peripheral parts of the atherosclerotic plaque are characterized by excessive deposition of the extracellular matrix.

Non-resolving inflammation plays a crucial part in the formation of the most dangerous unstable plaques that are prone to thrombogenesis (8). It was found that deletion of certain inflammatory genes leads to a reduction of atherosclerosis independently from changes of the circulating lipid levels (9). Mechanistically, thrombosis at the surface of an unstable plaque can be explained by the rupture of the plaque fibrous cap that has protective functions, and the exposure of the lipid-rich core of the plaque that contains tissue factors to the circulating blood. As a result, the coagulation cascade is activated, leading to platelet aggregation and thrombosis. However, about 30% of all thrombotic events are associated with intact atherosclerotic plaques that have only superficial endothelial erosion and are proteoglycan-rich (1,10).

Matrix metalloproteinases (MMPs) are secreted by macrophages and other inflammatory cells in the plaque. MMPs are responsible for matrix degradation that leads to collagen depletion in the fibrous cap of the plaque, and is typically associated with plaque rupture (11). Collagen depletion in the fibrous cap is associated with massive death of VSMCs that synthesize the extracellular matrix.

The exact mechanisms of plaque erosion remain unclear. Among the pathways responsible for this process, different authors have named local platelet-mediated neutrophil activation, release of myeloperoxidase, TLR-2 signaling in the endothelium, neutrophil-mediated injury, and apoptosis of endothelial cells. Neutrophils appear to play a special role in this process. Activated neutrophils can release their contents, including DNA and proteins, into the extracellular space forming the so-called neutrophil extracellular traps (NETs) during the process called NETosis, a special type of cell death. Formation of NETs was shown to contribute to atherosclerosis (12,13). In summary, atherosclerosis development involves a complex network of various cells and signaling pathways that may also vary from one stage of atherosclerosis development to another. Studying of these mechanisms requires equally complex tools, such as animal models that have already delivered a large amount of information that improved our understanding of atherosclerosis pathogenesis (14).

## Animal models of atherosclerosis

Common requirements for animal models of human diseases include compatibility with human anatomy and physiology, translational potential, relative ease of maintenance, and affordable cost. When modelling atherosclerosis, it is important that animal models share the topography of the lesions with that observed in humans. Reproduction of atherosclerosis features in animal models is based on accelerated plaque formation that can be achieved by different approaches. The most frequent methods include cholesterol-rich diets and modifications of genes involved in lipoprotein metabolism. Mice and rabbits remain the most common choice for atherosclerosis model creation, followed by pigs and non-human primates. This review aims to summarize the information on the most commonly used animal models of atherosclerosis and their specific features

Each of the models has both advantages and limitations. Murine models are characterized by a short life cycle, high reproduction rate, and simplicity of manipulation that makes their use convenient for modelling atherosclerosis (15). Rabbits are phylogenetically closer to humans than rodents, and rabbit genome sequencing and transcriptomic profiling of atherosclerosis have been successfully completed. These features make rabbits one of the most suitable species for studying atherosclerosis (16). Other advantages of rabbit models include the ease of manipulation, relatively low cost, short gestation period, large numbers of progeny, relatively suitable size, and short lifespan. Rabbits are often used for translational research such as pre-clinical testing of drugs and diagnostic methods for patients (17,18).

Genetically modified animals revolutionized the approaches to animal model creation in many disease areas, including atherosclerosis. Methods of introducing modifications to DNA through molecular manipulations are being constantly improved. Currently, it is possible not only to insert or inactivate genes of interest, but to create conditional knock-outs silencing certain genes in particular organs and tissues or in response to an external signal.

Currently, genetically modified mice are commonly produced using one of the two basic technical approaches. The first method is used to manipulate a single gene, for example for knocking out or single nucleotide changing. In this method, embryonic stem cells are modified with a DNA construct containing DNA sequences homologous to the target gene, and then injected into blastocysts (19). The other approach is used for insertion of the new genetic information into the mouse genome or for over-expression of certain endogenous genes. It involves pronuclear injection into a single cell of the mouse embryo, where it randomly integrates into the mouse genome (20). Many rodent transgenic models of atherosclerosis and associated conditions have already been developed and characterized, therefore in most cases, there is no need to design a model anew (Table 1).

**Table 1:** Overview of rabbit and mouse models of human atherosclerosis.

Animal model	Name	Main features	References
**Rabbit models**			
Watanabe heritable hyperlipidemic rabbits	WHHL rabbits	- spontaneously developing hypercholesterolemia and atherosclerosis on normal diet - 8-14-fold increased serum levels of cholesterol and triglycerides compared to normal Japanese white rabbits	(27-29)
Animal model for spontaneous myocardial Infarction (WHHLMI rabbit)	WHHLMI rabbits	- spontaneously developing hypercholesterolemia and atherosclerosis on normal diet - 8-14-fold increased serum levels of cholesterol and triglycerides compared to normal Japanese white rabbits - ability to form calcified plaques - acute myocardial infarctions	(30,31)
Apolipoprotein E knock-out rabbits	ApoE^-/-^ rabbits	- develop mild hyperlipidemia on normal diet - develop marked atherosclerosis on cholesterol diet	(26)
Lipoprotein (a) in transgenic rabbits	Lp(a)-rabbits	- develop atherosclerosis on cholesterol-rich diet - demonstrate special aspects of lipoprotein metabolism - lesions were shown to be significantly increased in the aorta, the iliac artery, and the carotid artery	(32)
**Mouse models**			
Apolipoprotein E knock-out mice	ApoE^-/-^ mice	- spontaneously developing atherosclerosis on normal diet - lesion progression, cell types present in the atherosclerotic plaque and presence of oxidized LDL reflect the situation observed in humans	(39,40,43-46)
LDL receptor-deficient mice	Ldlr^-/-^ mice	- milder lipoprotein profile alteration compared to ApoE^-/-^ mice - atherosclerotic lesions develop in time-dependent manner	(37,50,52)
PCSK9 adeno-associated virus mice	PCSK9 adeno-associated virus mice	- develop atherosclerosis on fat-rich diet - allow the study of plaque calcification	(53-57)
SR-BI knockout and ApoE-hypomorphic mice	SR-BI KO/ApoeR61h/h mice	- development of atherosclerosis and coronary heart disease on diet rich in fat, cholesterol, and cholate - formation of advanced plaques - severe coronary heart disease and even premature death seen in humans	(58,59)
apoE3Leiden.CETP mice	apoE3Leiden.CETP mice	- form all stages of atherosclerotic lesions in diet-induced manner - human-like response to treatment with such drugs as statins, fibrates, and ezetimibe	(61)
Apolipoprotein E-deficient fibrillin-1 mutant mice	ApoE^-/-^Fbn1C1039G^+/-^ mice	- resemble plaque rupture - resemble human-like complications	(62)

## Rabbit models of atherosclerosis

Rabbit models of atherosclerosis became less frequently used since 2000, when apolipoprotein E (apoE) and low-density lipoprotein (LDL) receptor knock-out mice were developed (17).

The popularity of rabbit models is explained by the fact that these animals are relatively inexpensive and easy to maintain (21). Lipoprotein metabolism in rabbits is comparable to that of humans, however, rabbits are characterized by a relative deficiency of hepatic lipase. In terms of lipid metabolism, rabbits are superior to mice for modelling the human situation, since in rabbits, significant amounts of cholesterol are present in apolipoprotein B-containing LDL and very low-density lipoprotein (VLDL), while in mice, the predominant plasma lipoprotein is high-density lipoprotein (HDL) (22,23). However, rabbit models of atherosclerosis have their limitations. One of them is massive inflammation and hepatic toxicity that develop in response to long-term cholesterol-rich feeding aimed to induce hypercholesterolemia (24). Nevertheless, rabbit models of atherosclerosis have been successfully used for more than 100 years, and allowed studying several fundamental disease mechanisms, including establishing the key role of elevated plasma cholesterol in atherosclerotic plaque formation (24). Modern techniques allowed creating more reliable rabbit models of atherosclerosis, such as Watanabe hereditary hypercholesterolemic animals (25), apolipoprotein E knock-out (ApoE^-/-^) animals (26), and diet-induced atherosclerotic New Zealand White rabbits (27).

## Watanabe heritable hyperlipidemic rabbits

The Watanabe heritable hypercholesterolemic (WHHL) rabbit line was established based on a mutation that causes a defect in the LDL receptor. Such animals are characterized by spontaneously developing hypercholesterolemia and atherosclerosis (25). Homozygous WHHL rabbits that are kept on a normal diet present with hypercholesterolemia from birth, with LDL being the predominant circulating lipoprotein. These rabbits develop various forms of atherosclerotic lesions, from early fatty streaks to advanced lesions in the aorta, coronary arteries, and cerebral artery (27). This rabbit model was one of the first models that allowed demonstrating the suppressive effect of statins on plaque destabilization and associated thrombogenesis (28). Furthermore, this model allowed investigating the effect of insulin resistance on atherosclerosis lesion formation due to early insulin resistance and glucose tolerance development in such animals. High-fructose and high-fat diet induced aortic lesions with a lipid core and calcifications in WHHL rabbits replicating the human situation (29). Moreover, these animals demonstrated spontaneous development of aortic atherosclerosis and myocardial infarction.

## Watanabe heritable hyperlipidemic rabbits for spontaneous myocardial infarction

Watanabe heritable hyperlipidemic rabbit model for spontaneous myocardial infarction (WHHLMI) was created in the attempt to further refine the WHHL model by selective breeding of myocardial infarction-prone animals for several years (30). The resulting animal had a high incidence (up to 97%) of fatal myocardial infarction caused by coronary atherosclerosis. Moreover, atherosclerotic plaques developing in these animals shared common features with human unstable plaques, such as a thin fibrous cap and the presence of a necrotic core. During recent years, WHHLMI rabbits were evaluated for studying human coronary atherosclerotic plaque initiation, formation, and development. Histopathological examination of 187 animals revealed various types of coronary atherosclerotic lesions, including fatty streaks, fibroatheromas, fibrous lesions, advanced lesions with calcification and signs of neovascularization, and lesions resembling human unstable plaques (31).

## Apolipoprotein E knock-out (ApoE^-/-^) rabbits

ApoE^-/-^ rabbits were designed as a model for investigating the relationship between atherosclerosis and human hyperlipidemia (26). These animals represent a promising alternative to *apoE* ^-/-^ mice, because of the better match of the rabbit lipoprotein profile to that of humans. Knocking out the ApoE gene was achieved by different research groups using a range of genome editing approaches, such as RNA-guided CRISPR-associated protein 9 (Cas9) endonucleases, zinc finger nucleases, and transcription activator-like effector nucleases (TALENs) methods. Even when kept on a normal diet, ApoE^-/-^ rabbits develop mild hyperlipidemia. Total cholesterol level in such animals remains about 200 mg/dL, and can be increased up to 1000 mg/dL upon feeding with a cholesterol-rich diet (0.3% cholesterol and 3% soybean oil) for two weeks, while wild-type rabbits fed with a cholesterol-rich diet only demonstrate a cholesterol level increase up to 170 mg/dL. Moreover, ApoE^-/-^ rabbits develop more pronounced aortic atherosclerosis than wild-type rabbits when fed a cholesterol-rich diet for 10 weeks. Due to important roles of both ApoE and LDL receptor in the regulation of cholesterol metabolism, using ApoE^-/-^ rabbits together with LDL receptor-deficient WHHL rabbits appears to be promising for modelling human hyperlipidemia (26).

## Lipoprotein (a) transgenic rabbits

The transgenic rabbit model expressing human apolipoprotein(a) was developed using white Japanese rabbits. Unlike rodents, rabbit apoB is capable of binding to recombinant human apo(a) forming lipoprotein(a) (Lp(a))-like particles in the plasma (32). Transgenic Lp(a) rabbits develop more pronounced atherosclerosis in response to a cholesterol-rich diet and also demonstrate other special aspects of lipoprotein metabolism. The lesions in such animals were shown to be significantly increased in the aorta, the iliac artery, and the carotid artery compared with normal rabbits, which makes this model illustrative for the investigation of some aspects of human atherosclerosis (32).

## Rodent models of atherosclerosis

Rodents have been a popular choice for developing atherosclerosis models since 1960s (33). Historically, the first murine model of atherosclerosis was diet-induced disease in mice fed with cholesterol and cholate-containing foods (34). However, rapid development of genetic engineering methods widely broadened the possibilities of creating murine models. Two *apoE* ^-/-^ mice strains were created independently by 2 research groups in the early 1990’s (35,36). An important feature of *apoE* ^-/-^ mice is the ability to develop atherosclerosis even while being fed standard rodent chow diets. Another commonly used murine model of atherosclerosis is LDL receptor-deficient (*ldlr* ^−/−^) mice that replicate human familial hypercholesterolemia (37). Currently, mice are the most frequently used animals in atherosclerosis research due to multiple advantages, from the ease in maintaining to the availability of numerous established variants of genetic modifications.

## Apolipoprotein E knock-out (apoE^-/-^) mice

In the blood plasma, apoE can be associated with chylomicron remnants, LDL, and HDL. It acts as a ligand for the hepatic uptake of chylomicron remnants and intermediate density lipoproteins by LDL receptors (LDLR) and the LDLR-related protein 1 (LRP-1) (37). The homology between human and mouse apoE reaches 70% (38). This protein has been identified as a promising target for creation of atherosclerosis models early on (35,36). Creation of *apoE* ^-/-^ mice was achieved by the replacement of the wild-type gene with a mutated variant that does not produce functional protein (39).

In *apoE* ^-/-^ mice, atherosclerosis development is initiated spontaneously, even when animals are kept on a regular rodent diet, but can be accelerated by applying cholesterol- and fat-enriched Western type diet. Atherosclerotic lesions typically develop in the aortic root, the aortic arch, innominate artery, carotid arteries, and other arteries, depending on the diet and the duration of cholesterol-rich feeding (40). A distinctive feature of this model is the rarity of lesion development in the carotid arteries. Lesion progression, cell types present in the atherosclerotic plaque, and presence of oxidized LDL in *apoE* ^-/-^ mice reflect the situation observed in humans. The murine model represents all stages of atherosclerotic lesion progression, but does not allow modelling the plaque rupture that occurs in humans (39).

Development of *apoE* ^-/-^ mice was an important step in the study of atherosclerosis and helped to establish some important disease mechanisms. In particular, the role of chronic inflammation in atherosclerosis initiation and progression was studied using this model (41). Moreover, *apoE* ^-/-^ mice are frequently used for testing potential therapeutic agents and environmental factors that may affect atherosclerosis development. For instance, this model allowed evaluating the effect of probucol on atherosclerotic development, which appeared to be paradoxical in *apoE* ^-/-^ mice and LDL receptor-deficient mice (42,43). Another study has evaluated the effect of dietary vitamin E supplementation in *apoE* ^-/-^ mice and demonstrated its beneficial effects on atherosclerosis development (44). Antiatherogenic effects of angiotensin-converting enzyme inhibitors or the angiotensin-II receptor antagonists were also studied using this model (45,46).

## LDL receptor-deficient mice (ldlr^-/-^)

LDL receptor (LDLR) is a plasma membrane protein that mediates the hepatic clearance of plasma lipoproteins containing apolipoproteins apoB100 or apoE (14). Mutations in the *LDLR* gene are associated with familial hypercholesterolemia in humans. Naturally, this protein appeared to be an attractive target for creating knock-out animals prone to atherosclerosis development. Mice lacking the *ldlr* gene (*ldlr* ^-/-^) were created using the gene targeting approach in 1994 (37). These mice are characterized by a milder lipoprotein profile alteration compared to *apoE* ^-/-^ mice, with a plasma cholesterol level being around 250 mg/dL on a regular diet (47). Without dietary induction, *ldlr* ^-/-^ mice develop atherosclerosis relatively slowly. However, application of high-fat and cholesterol diet can accelerate the process dramatically, with plasma cholesterol levels rising above 1500 mg/dL (37,48). In *ldlr* ^-/-^ mice, circulating cholesterol is present almost entirely in the LDL fraction, which replicates the human lipoprotein profile and can therefore be used for modelling purposes (49–51). In these animals, atherosclerotic lesions develop in a time-dependent manner, and the formation of plaques begins in the proximal aorta, later spreading to other arteries (52).

## PCSK9 adeno-associated virus mice

A novel murine model of atherosclerosis, PCSK9 adeno-associated virus mice, was developed without using germline genetic engineering (53). Designing of this murine model required only a single injection of a recombinant adeno-associated virus (AAV) containing PCSK9 gain-of-function mutant forms of PCSK9, human PCSK9D374Y or mouse PCSK9D377Y (AAVmPCSK9). These genes in combination with a high-fat diet were sufficient to reduce the LDLR expression, increase plasma LDL cholesterol, and induce atherosclerosis in mice or hamsters (54). Aortic root lesions developed in PCSK9 adeno-associated virus mice after the induction of atherosclerosis by a high-fat diet. Histological analysis of lesions showed advanced plaque development with foam cells, smooth muscle cells, and fibrous tissue present in the plaques (53). Moreover, this model allowed the study of plaque calcification (55,56). Diet-dependence is an important feature of this model, which also allows the study of atherosclerosis regression. It was shown that simply switching these mice to a regular diet for 6 weeks could induce lesion regression (57).

## SR-BI knock-out and apoE-hypomorphic mice

This model was generated by breeding two mice strains: SR-BI-deficient (SR-BI KO) mice and hypomorphic apoE mice (ApoeR61^h/h^): SR-BI KO/ApoeR61^h/h^ mice. The most significant feature of the SR-BI KO/ApoeR61^h/h^ mouse is the development of atherosclerosis and coronary heart disease in response to an atherogenic diet rich in fat, cholesterol, and cholate. It allows investigators to control the time of disease onset, and also the severity of symptoms (58).

This model is of specific interest due to the lack of small animal models resembling severe atherosclerosis symptoms (formation of advanced plaques), severe coronary heart disease, and even premature death seen in humans (59,60).

## ApoE3Leiden.CETP mice

The recently developed apoE*3‐Leiden.CETP (E3L.CETP) mouse model of atherosclerosis appears to be the one that most closely replicates the features of human disease. Among the similarities are the ability to form atherosclerotic lesions of all stages (type I to V) in a diet-induced manner and the response of diseased animals to the treatment with such drugs as statins, fibrates, and ezetimibe. The model was created by combining the apoE*3‐Leiden transgene that provides reduced clearance of triglyceride‐rich lipoprotein, and the cholesteryl ester transfer protein (CETP) transgene that makes the cholesterol profile more humanized (61).

## ApoE-deficient fibrillin-1 mutant (ApoE^-/-^Fbn1C1039G^+/-^) mice

These mice are characterized by impaired production of fibrillin-1, which is responsible for the fragmentation of elastic fibers observed in aortic stiffening. This feature is known to be a potential cause of plaque rupture. The model also shares the common features of atherosclerotic (*apoE* ^-/-^) mice, and can therefore be used to study the features of human unstable plaques. ApoE^-/-^Fbn1C1039G^+/-^ develop atherosclerosis in response to a high-fat diet, and this process is accelerated compared to regular *apoE* ^-/-^ mice (62).

## Conclusions

Animal models proved to be indispensable for studying human diseases, including atherosclerosis and searching for novel therapeutic approaches. Currently, several reliable rabbit and mice models of atherosclerosis have been developed and validated. Most of them are based on genetic modifications of key genes involved in atherosclerosis development, such as apolipoprotein E or LDL receptor genes. The models vary in terms of blood lipid profile, the ability to develop atherosclerotic lesions spontaneously or induced by a special diet, and the presence of complicated and unstable plaques. While induction of atherosclerosis lesions in model animals can be achieved reliably, modelling of complicated plaques, with such features as calcification, neovascularization, intraplaque hemorrhage, and thrombosis, is more challenging. Future studies should concentrate on creating such models that would allow testing new medications aimed at plaque stabilization.
